# Complete Genome Sequences of Mycobacteriophages Candle, Schatzie, Sumter, and Waleliano

**DOI:** 10.1128/MRA.00643-19

**Published:** 2019-07-25

**Authors:** Kayla M. Fast, Brianna E. Forrest, Garren R. Granec, John D. Larrimore, Anna E. Morse, Emma D. Ryan, P. Kiersten Schellhammer, Tracy W. Keener, Michael W. Sandel

**Affiliations:** a Department of Biological and Environmental Sciences, University of West Alabama, Livingston, Alabama, USA; DOE Joint Genome Institute

## Abstract

Mycobacteriophages Candle, Schatzie, Sumter, and Waleliano were isolated from soil using the host bacterium Mycobacterium smegmatis mc^2^155. Candle, Schatzie, and Sumter were discovered in Alabama and Waleliano in Maryland. The bacteriophages have been assigned clusters based on nucleotide similarity, as follows: Candle, R; Schatzie, J; Sumter, A1; and Waleliano, B4.

## ANNOUNCEMENT

It is estimated that bacteriophage particles are some of the most abundant biological entities on the planet (1). Four bacteriophages (phages) were discovered and the genomes annotated by University of West Alabama (UWA) students and faculty. The phages, designated Candle, Schatzie, Sumter, and Waleliano, were discovered in the United States (Table 1).

**TABLE 1:** Characteristics and accession numbers of bacteriophage genomes

Bacteriophage	GenBank accession no.	Genome size (bp)	No. of protein CDSs^*a*^	No. of tRNAs	G+C content (%)	Location; coordinates	Shotgun coverage (×)	Phage life cycle	Closest relative (accession no.), nucleotide identity (%), coverage (%)
Candle	MK757446	71,390	95	0	56.0	Tuscaloosa County, AL; 33.26, −87.61	564	Lytic	Send513 (JF704112), 99.26, 99.0
Schatzie	MK524521	111,345	232	1	60.8	Sumter County, AL; 32.59, −88.19	699	Lysogenic	Hughesyang (MK524504), 99.4, 89.0
Sumter	MK814754	52,656	90	0	63.7	Sumter County, AL; 32.36, −88.11	1,058	Lysogenic	Lockley (EU744249), 95.43, 83.0
Waleliano	MK524486	70,963	96	0	68.9	Baltimore County, MD; 39.25, −76.71	408	Lytic	BrownCNA (KT270441), 98.96, 99.0

a CDSs, coding sequences.

The host bacterium Mycobacterium smegmatis mc^2^155 was used in the isolation of these phages. M. smegmatis was cultured from a frozen glycerol stock with incubation in 7H9 medium at 37°C with shaking (250 rpm) for 5 days (2). For our purposes of characterizing and finding novel phages, this host is suitable because data are available for over 10,000 phages known to infect M. smegmatis (as of June 2019 [3]). Candle and Schatzie were isolated from soil samples at 37°C directly in 7H9 medium (2). Sumter and Waleliano were enriched with the host bacterium in addition to incubation in 7H9 medium. Imaging with a transmission electron microscope showed that all four phages belong to the family *Siphoviridae,* given the noncontractile tails, which ranged from 142.0 to 335.29 nm long, and with isometric heads ranging from 42.0 to 83.3 nm.

Purified high-titer lysates (HTLs) were collected from plaques, and the phage life cycles are given in Table 1. Whole-genomic DNA was extracted from HTLs using the Wizard DNA clean-up system (Promega, Madison, WI [2]). The NEBNext Ultra II FS kit (New England BioLabs, Ipswich, MA) with dual-indexed barcoding was used to build DNA libraries, which were then pooled for sequencing. Sequencing was performed at the Pittsburgh Bacteriophage Institute using an Illumina MiSeq platform. Each genome yielded at least 200,000 single-end 150-base reads; the coverage depths are listed in Table 1. Assembly was performed using Newbler 2.9, with default settings (4). This produced a single contig for each genome, which was used to determine genome ends and was checked for completeness and accuracy using Consed 2.0 (5). Two genomes exhibited defined ends with a 4-bp overhang (ATCC) in Schatzie and 10-bp overhang (CGGATGGTAA) in Sumter. Candle and Waleliano did not show a buildup of reads or coverage variation and were designated circularly permuted, as previously described (6). The beginning of each genome was chosen based on similar phages. The genome lengths and G+C contents are given in Table 1.

Genome annotation was performed in DNA Master 5.23.3 (http://cobamide2.bio.pitt.edu/computer.htm), and the Sumter genome annotation was supplemented with the Phage Evidence Collection and Annotation Network (PECAAN; https://pecaan.kbrinsgd.org/index.html). Gene function and start sites were determined using PhagesDB BLAST, NCBI BLAST, Phamerator (https://phamerator.org/), GeneMark 3.25, Starterator (https://github.com/SEA-PHAGES/starterator), and HHpred 3.0 (3, 7–9). Detection of tRNAs was performed in ARAGORN 1.2.38 and tRNAscan-SE 2.0 (10, 11). Protein-coding gene and tRNA totals are provided in Table 1. Each phage was assigned to a cluster, as follows: Candle, R; Schatzie, J; Sumter, A1; and Waleliano, B4 (12). Cluster assignment employs dotplot analysis in Gepard 1.30 and comparison of average nucleotide identities (13). Similarity to the closest relative of each phage was determined using NCBI BLAST (Table 1) (7).

### Data availability.

The complete genome sequences of Candle, Schatzie, Sumter, and Waleliano are available from GenBank under the accession numbers MK757446, MK524521, MK814754, and MK524486, respectively. Raw Illumina reads for Candle, Schatzie, Sumter, and Waleliano are available on NCBI’s Sequence Read Archive under accession numbers SRX5736299, SRX5736298, SRR8956689, and SRX5736300, respectively.
